# High Technology in Medicine: Lessons from Cardiovascular Innovations and Future Perspective

**DOI:** 10.5041/RMMJ.10109

**Published:** 2013-04-30

**Authors:** Rafael Beyar

**Affiliations:** Rambam Health Care Campus and the Technion-Israel Institute of Technology, Haifa, Israel

**Keywords:** Angioplasty, drug-eluting stents, induced pluripotent cells, percutaneous aortic valves, percutaneous coronary interventions, stents

## Abstract

Four decades of innovations in the field of interventional cardiology are presented as an example for the great growth of high technology in medicine, side by side with the development of general technology and science. The field of percutaneous coronary intervention (PCI) was enabled by the development of X-ray systems, allowing us to view the pathology, and was critically dependent on courageous and imaginative physicians and scientists who developed percutaneous transluminal coronary angioplasty (PTCA), stents, and transarterial aortic valve replacement (TAVR). Today, outstanding research continues to progress, with stem cell research and IPC technologies presenting new challenges and yet taller mountains to climb. The rapid development we have witnessed was due to tight collaborations between clinical and academic institutions and industry. The combination of all these elements, with a proper mechanism to handle conflict of interest, is an essential linkage for any progress in this field. We will continue to see exponential growth of innovations and must be prepared with appropriate bodies to encourage such developments and to provide early-stage funding and support for novel ideas.

## INTRODUCTION

In the last 50 years our technological abilities have expanded in an unprecedented way and have undergone several phases that have dramatically changed our lives. Advances have been made in material sciences, chemical analysis, physics and imaging, communications, energy transmission, miniaturization of devices and material structures, and nanotechnology. Our understanding of the molecular mechanisms of disease, along with the ability to design complex molecules, has expanded the development of new drugs and therapeutic modalities. Deciphering the genome and its function has enabled enhanced diagnostics and therapeutics, and has paved the way for unprecedented control of the genomic structure that is applied today to plants and experimental models involving single cell life forms, as well as complex animals.

All of these technologies are being applied to medicine in the search for a better understanding and cure of diseases. Novel scientific discoveries achieved via on-going basic research has led to the expansion of human knowledge and a better understanding of the basic processes involved in life and disease. Translational research that takes advantage of this new knowledge and applies it to diagnose and cure disease has proliferated in the constant search for better ways to treat our patients. This paper examines the impact of our novel technologies on developments in the medical field, with a special window on cardiovascular interventions and the mechanisms applied for this unprecedented progress via technology.

## THE BIRTH OF CATHETERIZATION AND THE DEVOTION OF YOUNG INVESTIGATORS

Clinical giants with a daring spirit led to our current practice in cardiovascular medicine. With the major discovery of X-ray imaging in 1895 by Wilhelm Conrad Röntgen, who was awarded the first Nobel Prize in Physics in 1901,^1^ the human body became transparent for the first time, and we could look into it without having to cut it open. However, application to the cardiovascular discipline took more time. Werner Forssmann was a young and passionate physician from Edelweiss, Germany. In 1929 he dared to introduce a ureteric catheter through the antecubital vein of his own arm towards his heart.^2^ To do so, he had to constrain the nurse to the catheterization table. He then imaged his heart with the X-ray system and saw that the catheter was placed in the right atrium. In his paper he suggested that such catheters could be used to measure pressures in the heart chambers and inject radiopaque dye. It took another 26 years before this diagnostic method became widely recognized, and, together with Andre Cournand and Dickinson Richards, he received the Nobel Prize in 1956.^3^ Shortly thereafter, in October of 1958, coronary angiography was suggested by Mason Sones who accidentally injected contrast dye into the coronary artery via a catheter placed in the aorta of a patient undergoing heart catheterization. The patient experienced a cardiac arrest but survived. That finding led to the development of coronary angiography, and coronary artery disease could be seen and characterized for the first time in living patients.^4^ With this powerful diagnostic tool at hand, the field of cardiac bypass surgery was born; Robert Goetz performed the first venous bypass graft and published his results in 1961.^5^ Bypass surgery has proliferated since then and undergone years of uncontrolled expansion for a variety of clinical indications. It subsequently shrank back to smaller and steady numbers based on the cumulative evidence generated in major studies. Bypass surgery became the standard of care for multi-vessel and left main revascularization procedures and remains valid to this day.

## A CLINICALLY DRIVEN PASSIONATE INNOVATOR—THE BIRTH OF INTERVENTIONAL CARDIOLOGY

Percutaneous transluminal coronary angioplasty (PTCA) was the next frontier challenging the surgical methods for coronary revascularization. The concept of transluminal angioplasty was suggested by Charles Dotter as early as 1964.^6^ Dotter pioneered modern medicine with the invention of angioplasty, which was first used to treat peripheral arterial disease. Dotter is commonly known as the “Father of Interventional Radiology” and was nominated for the Nobel Prize in Medicine in 1978. Dr Andreas Grüntzig followed Dotter’s concept in 1974 and performed the first peripheral human balloon angioplasty.^7^ However, he did not stop there. Grüntzig hypothesized that coronary blockages can be dilated by a balloon in an alert patient and that the artery will remain open after that. He achieved his goal by building some experimental balloons on long catheters from plastic materials available at that time. In 1977 he treated the first patient with this technique and dilated a proximal lesion at the left anterior descending artery.^8^ The patient recovered and that artery remained open for many years. The balloon that Grüntzig developed looks exactly like the balloons used today. The field of interventional cardiology was born by the passion of a physician who carried his idea to the patient’s bedside.

That technology, broadly known today as percutaneous coronary intervention (PCI), sparked a lot of criticism. In the early days of angioplasty, the dilated artery would close abruptly in up to 10% of patients, leading to mortality in over 30% of those patients. In addition, restenosis occurred within 3 months in over 30% of the patients due to a combination of vessel recoil and intimal proliferation, in response to the injury caused by balloon dilatation. Over the years, materials have improved and the thinner profile of newer catheters allowed less traumatic interventions. Nevertheless, balloon dilatation continued to be limited by acute occlusion and restenosis, necessitating the search for appropriate solutions.

In summary, Andreas Grüntzig, an enthusiastic, passionate, and talented physician who was inspired by earlier pioneers, was able to solve technological and conceptual barriers and apply his solution to patients bravely, in the face of much criticism. His work gave birth to a fascinating new world and opened the door for the influx of new technologies for years to come. He died in 1985 in a plane accident, but the field that he inspired has grown beyond his expressed dreams.

## WHEN A PHYSICIAN MEETS AN ENGINEER OR HAS ENGINEERING IN HIS BLOOD

The search for a solution to the problems of acute arterial occlusion immediately after balloon dilations and long-term restenosis has sparked many engineering attempts in the first decade of PCI. Various drilling devices, applied energy such as hot balloons, lasers, and other methods have been tried; however, none of these techniques showed a significant benefit. Metal stents were proposed by several groups as a method to scaffold the weak and irregular surface of the arterial wall at the stenosis site. The early days of stenting were fascinating. Jacques Puel and Ulrich Sigwart implanted the first coronary stent in humans—the wallstent—in 1986 in Toulouse, France.^9^ This stent never received Food and Drug Administration (FDA) approval for coronary applications. The first coronary stent that was approved by the FDA, in 1993, was the Gianturco–Roubin stent.^10^ The Palmaz–Schatz stent was approved the following year.

The story of the Palmaz–Schatz stent emphasizes the tight interaction between engineers and physicians and reveals how an invention is born from a conceptual model. Julio Palmaz was a young physician who came to the USA to pursue research. He had an idea for a metal structure designed to hold the artery open. First he had to find the right material. One of his first choices was copper, bought at a RadioShack store. However, he soon recognized this was the wrong metal, as it produced intense inflammation and restenosis. The optimal choice turned out to be medical-grade stainless steel, with a more stable structure and only a limited inflammatory response on the arterial wall. An expandable slotted tube was developed and mounted on a balloon. Julio Palmaz was joined by a cardiologist, Richard Schatz; together they developed the first coronary stent that was entered in the pivotal clinical trial which led to FDA approval—two 7-mm long slotted tubes connected by a bridge. The bridge was critical for allowing some flexibility and permitting the stent to pass through the tortuous coronary artery. This stent, together with several early designs, pioneered the world of stenting. An animal model was a mandatory requirement, with canine or swine models being used in most cases.^10^

The early days of stenting were adventurous, with an initially high rate of early stent thrombosis.^9^,^11^ It took several years to understand the mechanism of this severe complication; eventually stent thrombosis would be prevented by combining full stent apposition to the vessel wall using high-pressure balloons with the use of potent antiplatelet drugs. The Palmaz–Schatz stent received FDA approval in 1994,^12^ greatly impacting this field, with additional stent designs applied to patients shortly after. More flexible stents with novel design such as the BeStent^13^ and the Nir stent^14^ were developed, and various metal surface modifications were applied to give the best clinical results. Newer metals such as nitinol, a nickel and titanium alloy with thermal memory, were used to generate self-expanding stent technologies that applied the appropriate strength at body temperature.^15^ All these developments resulted from tight collaborations between physicians and engineers with industrial and financial support around them. Many new companies were founded and later merged into larger companies. It was a bubbling and vibrant community with tight collaborations between academia, clinical institutes, and industry.

After FDA approval of the Palmaz–Schatz stent, stent penetration into the market was unprecedented. Within 4 years (1994–1998), stent usage climbed from 0% to 80% of PCIs. Abrupt coronary occlusion was minimized to a reasonable percentage, and restenosis was reduced (but not eliminated).

In a recent interesting paper, Xu et al.^16^ studied the innovative process in coronary stent development. Their results showed the central role of physician-innovators and their small private companies in helping create this field. Larger public companies made their contributions later in the product development time-line. The authors suggest implementing new policies in academic and clinical institutions, aimed at encouraging transformative medical device development through translational research at the early stages of technology development.

## THE TRIANGLE OF COLLABORATIONS BETWEEN INDUSTRY, ACADEMIA, AND PRACTICING PHYSICIANS

The disrupting technology of balloon angioplasty and stenting has driven numerous competitive attempts to develop stents from different metals such as tantalum, titanium, self-expanding nitinol alloy, and even gold coated with diamond dust.^17^ It has been a virtual parade of large and small industry-driven initiatives, attempting to improve this disruptive technology in small additive steps. Various manufacturing techniques involved major industries that specialized in stent-related technologies. Refining stent-balloon delivery performance and dealing with profile, flexibility, and tractability were huge challenges for this dynamic engineering world. Surface coating with inherent materials such as carbon, stable polymers, and even conjugated heparin molecules was attempted in order to achieve better tissue compatibility. However, restenosis was not reduced until the industry, sparked by combining pharmacology and biomaterials, developed the first drug-eluting stent. The first drug-eluting stent was a standard metal stent, coated with a layer of durable polymer containing sirolimus, an anti-proliferative drug, covered by another layer of polymer to control the release of the drug over 8 weeks.^18^

This represented a huge disruptive technology—an optimally matched combination of a device and a drug. It was also a victory for the tight collaboration between the engineers and scientists, appropriately applied to patients by clinicians. This classic triangle of interaction between industry, academia, and practicing physicians was once again proven successful.

Absorbable stents is another example of a concept driven by the combination of industry scientists and academic physicians.^19^ The development of a product and its refinement over several years started from a very few devoted clinicians who believed that a scaffold that dissolves over several months is better than a metal stent that becomes part of the arterial wall, persisting throughout the patient’s life. A similar approach, only with a metal, has been done with an absorbable magnesium stent.^20^ Only time will tell whether this technology will have additional benefit for patients.

The valvular revolution that we are witnessing today is another example of very intense developments involving all corners of the triangle. With the ability to implant an aortic stent via catheterization, transarterial aortic valve replacement (TAVR) was conceived by physicians and is currently applied to high-risk patients with aortic stenosis.^21^ This ability was made possible by refining and combining metal stent and biological valve technologies. It is an amazing tool, and currently aortic stent interventions are at a rapid expansion phase with proven evidence by large controlled randomized studies. The success in aortic stent devices stimulated and triggered multiple attempts to expand the horizon to new frontiers in the mitral space.^22^ Again, as in the early stent era, we see a plethora of innovative ideas, using the model of new startup companies that always involved a combination of passionate physician-scientists and a strong and capable engineering core.

## STEM CELLS AND BEYOND

The area of human embryonic stem cell technology was introduced by Thomson et al.^23^ in 1998, through a collaborative effort between the University of Wisconsin and academic work performed at Rambam Health Care Campus and the Technion in the Laboratory of Joseph Itskovitz. The first human stem lines in the world are therefore the outcome of an outstanding collaboration between academia (Technion and the University of Wisconsin) and a clinical hospital (Rambam Health Care Campus). This work was followed by an explosive growth in the field worldwide, stirring a plethora of ethical concerns among countries, societies, politicians, and religious bodies. Even established government research bodies such as the NIH had to apply ethical rules imposed on them by political leaders.

Despite these limitations, this field was vibrant with activity. Differentiation into cardiac cells was shown by Kehat et al. from the Technion and Rambam Hospital,^24^ and others have also shown differentiation into nerve and other cell types. Fueled by objections and debate, this field has generated much enthusiasm and hope for curing cardiovascular, neurological, metabolic, and other diseases. It has also reached a phase of early pilot clinical studies in several applications; however, to date, it has not shown a clear and proven benefit. The induced pluripotent cells (IPC) introduced by Yamanaka’s group, using genetic modification of adult fibroblasts,^25^ have been adopted in the Technion and Rambam and other laboratories as a potential solution to the ethical and immunological problems inherent to the human embryonic stem cell lines. Close to 15 years after the first paper on human embryonic stem cells in science,^23^ this field remains very active and stimulating and is currently driven by academia with very few attempts by industry to adopt it. Industry is hesitant both to invest the large sums required and to take the major risk involved in this therapeutic modality. While stem cells are a very strong scientific tool, without industry joining in, no progress will be achieved in applying this fascinating technology to our patients.

## PRINCIPLES OF COLLABORATIONS

**Figure 1.**
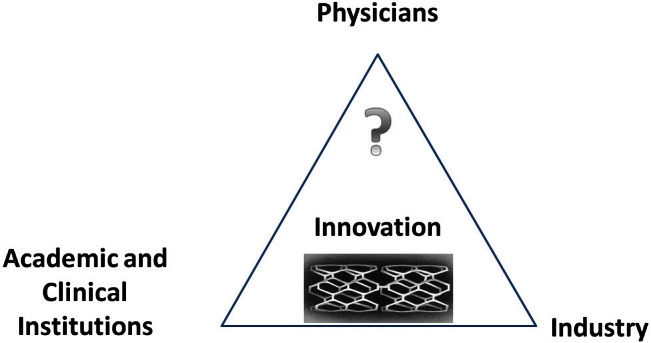
The triangle of collaboration.

As shown above, it is clear that, to advance medicine for the benefit of our patients, we must bring scientific ideas through technological progress. As history has shown, such advancement relies on intense collaboration between universities and academic hospitals, industry, and clinicians, independently of the source of the idea and who owns the intellectual property rights. This triangle of collaboration is schematically shown in Figure 1. The Palmaz–Schatz stent is illustrated as an example of a disruptive technology that changed medical practice.^11^ Obviously, we find the physicians and innovators, Julio Palmaz and Richard Schatz, at the top corner of the triangle. Several universities and hospitals were part of the clinical pathway to approval. The major industry that took the right corner is Johnson & Johnson. Without such collaborations this innovation would never have become reality. A scientist aiming at expanding human knowledge may identify a new mechanism that leads to a new therapy. However, he needs industry to join in and advance that mechanism towards a drug or a device, together with the physician who understands the clinical needs. A clinician who treats patients identifies an unmet need and explores ways to overcome this gap with industry, an academic scientist, or an entrepreneur-engineer. A company that seeks to expand its product line often evaluates ideas generated by these two sources, but also defines and develops new technologies in-house. As a rule, such industries rely on strong advice from their clinical consultants.

Therefore, to advance innovations we need to generate more collaboration methods, with built-in funding mechanisms for early ideas. This must happen within institutions, within countries, and between countries, in the form of international consortia that involve all three elements. Funding at the very early phases is highly important, so that these early seeds grow rather than dry up with time.

Within the academic and clinical institutions we need to secure the following mechanisms:

- A mechanism for exploring novel ideas and advancing them from the bench to the bedside.^16^ This involves creating specific funds to promote early research, and protection of the intellectual properties of staff members, while allowing for the high-quality publications mandatory for academic promotion, and finding pathways for collaborations with industry.
- A mechanism to deal with the conflict of interest that naturally exists when a medical device or a drug reaches the clinical study phase.^26^ This involves the combination of an appropriate institutional committee with full transparency of the investigator’s ties to the specific technology, to the patient, and to society. Such mechanisms exist in leading institutions worldwide and are a must in any institution conducting clinical research.

## THE ACADEMIC TRANSLATIONAL SCIENTIST

While it is agreed that science leads to progress in medicine, there are ample differences between basic and translational research, as discussed by Barry Coller.^27^,^28^Table 1 lists the key differences between a basic and a translational scientist.

**Table 1.** Translational versus basic research.

	Basic Research	Translational Research
Goal	Seek new knowledge	Improve human health
Way of Operation	Challenge accepted paradigms	Match discovery to clinical need
Methods	Designs experiments that disprove current hypothesis	Methodological pathway to proof of concept, proof of clinical benefit, and regulatory approval
Outcome	Novel data are added	New therapy or diagnosis is added

Basic scientists seek to add new knowledge and make discoveries. They test the validity of current conceptual models, challenge accepted paradigms, and design experiments that will lead to new mechanistic information that will transform the conceptual model in their discipline. This can eventually lead to many new applied therapeutic methods, but it is not an essential part of it. The best example that comes to mind is that of the Nobel Laureates, Avram Hershko, Aaron Ciechanover, andIrwin Rose,^29^ who discovered ubiquitin, the energy-dependent protein degradation system. Only 30 years later this new knowledge was translated to the bedside, and a drug against multiple myeloma (Velcade™ (bortezomib)) was developed based on the discovery of the ubiquitin pathway mechanism.^30^

Translational research scientists seek to improve human health by matching a discovery to a clinical need. The experiments that are required may involve both scientific and translational hypotheses. Bilateral bench and bedside experiments are needed, and often a few cycles and phases of such experiments are required. The ultimate outcome is a new therapy or diagnostic method, with proven benefit to the patient, based on a well-conducted clinical study, leading to regulatory approval and medical usage.

Translational scientists must have a conceptual understanding of the entire process leading to approval. They must be able to articulate a health need combined with a basic science hypothesis, to design a robust and tractable assay, and to conceptualize a pivotal study for proof of hypothesis leading to approval. They may do this alone, but it is better achieved with an expert group.

## PERSPECTIVES INTO THE FUTURE

It is clear that technology and science will continue to drive medicine through national and international collaborations. In just 40 years we will live to the age of 100. In the cardiovascular area, as in the majority of surgical or minimally invasive interventional disciplines, devices will control our clinical world, and our surgical abilities via small orifices will be enhanced. Our clinical world will be governed by information technology and mathematical predictions, whether an entire community, a hospital, or a single patient is involved. Genetics and genomics, analyzed by robust internet-based programs that will reside in a cyber-cloud, will become an integral part of our world and will govern our clinical decisions. Medical devices combined with imaging will continue to evolve and offer new therapeutic options. Combinations of a device and a drug eluted over the right time and in the right space through microchip mechanisms will be developed. Robotic and remote catheterization technologies will continue to evolve and introduce precision into the manually operated world.^31^–^33^ Surgery will be completely transformed to become minimally invasive and robotically driven, eliminating the need for large incisions. Genetically oriented molecular and cellular therapies will eventually beat cancer. As we reach the limit of our society to pay for medical care, cost sensitivity will remain a major factor in the development and wide availability of new devices and new therapeutics.

## Abbreviations:

FDA: Food and Drug Administration;
IPC: induced pluripotent cells;
PCI: percutaneous coronary intervention;
PTCA: percutaneous transluminal coronary angioplasty;
TAVR: transarterial aortic valve replacement.

## References

[1] The Nobel Prize in Physics 1901. Nobelprize.org. 23 Mar 2013. Available at: http://tinyurl.com/Nobel1901

[2] Forssmann W (1929). Die Sondierung des rechten Herzens. Klin Wschr.

[3] Bolt W, Knipping HW (1956). Congratulations to Werner Forssmann on winning the 1956 Nobel Prize for medicine. Med Klin (Munich).

[4] Proudfit WL, Shirey EK, Sones FM (1966). Selective cine coronary arteriography. Correlation with clinical findings in 1,000 patients. Circulation.

[5] Goetz RE, Rohman M, Haller JD, Dee R, Rosenak SS (1961). Internal mammary-coronary artery anastomosis. A nonsuture method employing tantalum rings. J Thorac Cardiovasc Surg.

[6] Dotter CT, Judkins MP (1964). Transluminal treatment of arteriosclerotic obstruction. Description of a new technic and a preliminary report of its application. Circulation.

[7] Grüntzig AR, Hopff H (1974). Perkutane Rekanalisation Chronischer Arterieller Verschlusse mit einem neuem Dilatasionskatheter. Dtsch Med Wocheschr.

[8] Grüntzig AR, Senning Å, Siegenthaler WE (1979). Nonoperative dilatation of coronary-artery stenosis — percutaneous transluminal coronary angioplasty. N Engl J Med.

[9] Sigwart U, Puel J, Mirkovitch V, Joffre F, Kappenberger L (1987). Intravascular stents to prevent occlusion and restenosis after transluminal angioplasty. N Engl J Med.

[10] King SB (1998). The development of interventional cardiology. J Am Coll Cardiol.

[11] Schatz R, Bairn D, Leon M (1991). Clinical experience with the Palmaz-Schatz coronary stent. Initial results of a multicenter study. Circulation.

[12] Kaplan AV, Baim DS, Smith JJ (2004). Medical device development: from prototype to regulatory approval. Circulation.

[13] Roguin A, Grenadier E, Markiewicz W (1999). One year clinical follow-up with the serpentine balloon expandable stent: report of the first 100 patients. Coron Artery Dis.

[14] Baim DS, Cutlip DE, O’Shaughnessy CD (2001). Final results of a randomized trial comparing the NIR stent to the Palmaz-Schatz stent for narrowings in native coronary arteries. Am J Cardiol.

[15] Beyar R, Henry M, Shofti R (1994). Self-expandable nitinol stent for cardiovascular applications: canine and human experience. Cathet Cardiovasc Diagn.

[16] Xu S, Avorn J, Kesselheim AS (2012). Origins of medical innovation: the case of coronary artery stents. Circ Cardiovasc Qual Outcomes.

[17] Serruys P (1996). Handbook of Coronary Stents.

[18] Sousa JE, Costa MA, Abizaid AC (2001). Sustained suppression of neointimal proliferation by sirolimus-eluting stents: one-year angiographic and intravascular ultrasound follow-up. Circulation.

[19] Ormiston J, Serruys PW, Regar E (2008). A bioabsorbable everolimus-eluting coronary stent system for patients with single de-novo coronary artery lesions (ABSORB): a prospective open-label trial. Lancet.

[20] Erbel R, Di Mario C, Bartunek J (2007). Temporary scaffolding of coronary arteries with bioabsorbable magnesium stents: a prospective, non-randomized multicenter trial. Lancet.

[21] Cribier A, Eltchaninoff H, Bash A (2002). Percutaneous transcatheter implantation of an aortic valve prosthesis for calcific aortic stenosis: first human case description. Circulation.

[22] Chiam PL, Ruiz CE (2011). Percutaneous transcatheter mitral valve repair. A classification of the technology. JACC Cardiovasc Interv.

[23] Thomson JA, Itskovitz-Eldor J, Shapiro SS (1998). Embryonic stem cell lines derived from human blastocysts. Science.

[24] Kehat I, Kenyagin-Karsenti D, Snir M (2001). Human embryonic stem cells can differentiate into myocytes with structural and functional properties of cardiomyocytes. J Clin Invest.

[25] Takahashi K, Tanabe K, Ohnuki M (2007). Induction of pluripotent stem cells from adult human fibroblasts by defined factors. Cell.

[26] Lo B, Wolf LE, Berkeley A (2000). Conflict-of-interest policies for investigators in clinical trials. N Engl J Med.

[27] Coller BS (2008). Translational research: forging a new cultural identity. Mt Sinai J Med.

[28] Coller BS (2012). Translating from the rivers of Babylon to the coronary bloodstream. J Clin Invest.

[29] Giles J (2004). Chemistry Nobel for trio who revealed molecular death-tag. Nature.

[30] Ciechanover A (2012). Intracellular protein degradation: from a vague idea through the lysosome and the ubiquitin-proteasome system and onto human diseases and drug targeting. RMMJ.

[31] Schmidt B, Chun KRL, Tilz RR, Koektuerk B, Ouyang F, Kuck KH (2008). Remote navigation systems in electrophysiology. Europace.

[32] Beyar R, Gruberg L, Deleanu D (2006). Remote-control percutaneous coronary interventions: concept, validation, and first-in-humans pilot clinical trial. J Am Coll Cardiol.

[33] Weisz G, Metzger DC, Caputo RP (2013). Safety and feasibility of robotic percutaneous coronary intervention. PRECISE (Percutaneous Robotically-Enhanced Coronary Intervention). J Am Coll Cardiol.

